# An Interplay among FIS, H-NS, and Guanosine Tetraphosphate Modulates Transcription of the *Escherichia coli cspA* Gene under Physiological Growth Conditions

**DOI:** 10.3389/fmolb.2016.00019

**Published:** 2016-05-24

**Authors:** Anna Brandi, Mara Giangrossi, Anna M. Giuliodori, Maurizio Falconi

**Affiliations:** Laboratory of Genetics, School of Bioscience and Veterinary Medicine, University of CamerinoCamerino, Italy

**Keywords:** *cspA* gene, FIS, H-NS, guanosine tetraphosphate, DNA-protein interaction, gene regulation in Bacteria, transcription

## Abstract

CspA, the most characterized member of the *csp* gene family of *Escherichia coli*, is highly expressed not only in response to cold stress, but also during the early phase of growth at 37°C. Here, we investigate at molecular level the antagonistic role played by the nucleoid proteins FIS and H-NS in the regulation of *cspA* expression under non-stress conditions. By means of both probing experiments and immunological detection, we demonstrate *in vitro* the existence of binding sites for these proteins on the *cspA* regulatory region, in which FIS and H-NS bind simultaneously to form composite DNA-protein complexes. While the *in vitro* promoter activity of *cspA* is stimulated by FIS and repressed by H-NS, a compensatory effect is observed when both proteins are added in the transcription assay. Consistently with these findings, inactivation of *fis* and *hns* genes reversely affect the *in vivo* amount of *cspA* mRNA. In addition, by means of strains expressing a high level of the alarmone guanosine tetraphosphate ((p)ppGpp) and *in vitro* transcription assays, we show that the *cspA* promoter is sensitive to (p)ppGpp inhibition. The (p)ppGpp-mediated expression of *fis* and *hns* genes is also analyzed, thus clarifying some aspects of the regulatory loop governing *cspA* transcription.

## Introduction

The *cspA* gene of *Escherichia coli* encodes a nucleic acid-binding protein of 70 amino acid residues (CspA) that interacts preferentially with single stranded RNA and DNA (Jiang et al., 1997; Bae et al., 2000). CspA belongs to the *csp* gene family, a group which includes in *E. coli* a total of nine paralogs, called from *cspA* to *cspI*. CspA is known as the “major cold-shock protein” (Goldstein et al., 1990; Jones and Inouye, 1994) by virtue of the original observation that cold-shock induces its synthesis *ex novo*. However, CspA is also synthesized at 37°C, particularly during the early phase of growth (Brandi et al., 1999; Brandi and Pon, 2012). In addition to *cspA*, also *cspB, cspE, cspG, cspI* are cold shock inducible, unlike *cspD, cspC, cspF* and *cspH* which are expressed only at 37°C. In particular, *cspD* is expressed exclusively during stationary phase or nutritional stress, *cspC* is constitutively synthesized at 37°C, and *cspF* and *cspH*, the most distant related genes, remain to be characterized (Jones and Inouye, 1994; Yamanaka et al., 1998; Ermolenko and Makhatadze, 2002).

During cold-shock, CspA was found to affect both transcription (La Teana et al., 1991; Jones et al., 1992b) and translation (Brandi et al., 1996; Giuliodori et al., 2004) of other genes and it was also suggested to function as an RNA chaperone (Jiang et al., 1997; Bae et al., 2000). On the other hand, little is known about the role played by CspA at 37°C. The expression of *cspA* displays a multilevel regulation (transcription, mRNA stability and translation) modulated by multiple factors that may differently contribute to ensure a rapid and precise response to nutritional or environmental changes (Gualerzi et al., 2011). Interestingly, the regulation of *cspA* expression is different under stress and non-stress conditions. In fact, while the elevated production of CspA following a temperature down-shift is due mainly to post-transcriptional events (i.e., increased stability and preferential translation of *cspA* mRNA) rather than to the transcriptional stimulation of *cspA* promoter (Brandi et al., 1996; Goldenberg et al., 1996; Giuliodori et al., 2010), the synthesis of CspA at 37°C is prevalently regulated at transcriptional level (Brandi et al., 1999; Brandi and Pon, 2012).

Bacteria contain a heterogeneous group of polypeptides, collectively known as nucleoid-associated proteins (NAPs), that are cataloged as a family on the basis of functional similarities. These proteins bind to nucleic acids, are basic and have low molecular mass (Azam and Ishihama, 1999). In addition to their common architectural role in the organization of bacterial chromosome, they are able to modulate transcription initiation and to control DNA replication, segregation and repair (Browning et al., 2010; Dillon and Dorman, 2010; Rimsky and Travers, 2011; Wang et al., 2011). H-NS, one of the most abundant NAPs, preferentially binds to tracts of intrinsically curved DNA (A/T-rich sequences) and/or actively induces bending (Yamada et al., 1990; Spurio et al., 1997; Gordon et al., 2011). Thus, by interacting with curved DNA, often found in upstream promoter regions, this nucleoid protein, referred to as a “universal repressor,” silences transcription of its target genes (Atlung and Ingmer, 1997; Hommais et al., 2001; Dorman, 2004, 2007; Bouffartigues et al., 2007; Lang et al., 2007). FIS (Factor for Inversion Stimulation) is another NAP that binds to DNA and modulates the topology of DNA in a growth-phase dependent manner (Schneider et al., 1997; Muskhelishvili and Travers, 2003). Unlike H-NS, FIS is a positive regulator activating transcription of genes and operons associated with primary metabolism as stable RNAs (Ross et al., 1990; Gonzalez-Gil et al., 1996). Thus, H-NS and FIS, through direct and indirect effects, control the expression of a large number of genes and are viewed as global regulators of transcription in response to growth phase and environmental changes (reviewed in Kahramanoglou et al., 2011).

Since *cspA* belongs to the set of genes controlled by FIS and H-NS under non-stress conditions (Brandi et al., 1999), the prime aim of this study was to elucidate the molecular aspects of *cspA* regulation by these two NAPs expanding and deepening our previous knowledge. Our results demonstrate the existence of a functional interplay between FIS and H-NS, which are able to bind separately or simultaneously the *cspA* promoter region. The composition of the DNA-protein complexes thus formed has a different impact on the transcription of *cspA*, that is inhibited by H-NS, stimulated by FIS, and unaltered when both factors are present, in fully agreement with the *in vivo* data (Brandi et al., 1999). Finally, seeking further factors which could participate to this regulatory circuit, we found that *cspA* promoter is sensitive to (p)ppGpp, the mediator molecule of stringent response.

## Materials and methods

### Strains

*E. coli* strains used in this study were: MRE600 (*F-, rna*) (Cammack and Wade, 1965); DH5α (Sambrook and Russell, 2001); WM2482 (correspond to MG1655 reference strain K-12, F^−^ λ^−^ *ilvG*^−^ *rfb-50 rph-1*), WM2648 (MG1655 *hns::hyg*), WM2649 (MG1655 *fis::kan*) and WM 2650 (*hns/fis* double mutant of MG1655) a kind gift of Walter Messer's laboratory (Berlin) (Afflerbach et al., 1998); *E. coli* KT793 carrying IPTG-inducible *relA* in pKT31 (Tedin et al., 1995). Cells were grown at 37°C in Luria-Bertani broth or in M9 minimal medium supplemented with 0.4% glucose (Sambrook and Russell, 2001) or in “Phosphates-free” medium (100 mM Tris-HCl, pH 7.7, 0.5% glucose, 0.5% peptone, 10 mM NH_4_Cl, 0.7 mM NaNO_3_, 1mM Na_2_SO_4_, 0.5 mM MgSO_4_·7H_2_O, 0.05 mM MnCl_2_·4H_2_O, 0.02 mM FeSO_4_·7H_2_O) where indicated.

### DNA manipulations and general procedures

The plasmid pTZ310 was constructed by cloning the HpaII-HpaII fragment, containing the *cspA* promoter region (from pos. −145 to pos. +165), into the AccI site of the pTZ19R polylinker. H-NS was purified as described in Falconi et al. (1988); FIS was a kind gift from the laboratory of Regine Kahmann. DNA isolation, agarose gel electrophoresis, polymerase chain reaction and other DNA manipulations were performed according to standard procedures (Sambrook and Russell, 2001). Radioactivity associated to DNA or RNA was detected and quantified by Molecular Imager (Bio-Rad, model FX).

### Northern blot analysis

Total RNA was purified by hot phenol extraction from cells harvested at the indicated times and levels of individual mRNAs were detected by Northern blots probed with specific 5′-end-labeled oligonucleotides (Brandi et al., 1999). The hybridization was performed in the range of temperature 37–48°C, depending on the oligonucleotide used. The oligonucleotides used as specific probes are: 5′-CTTTCGATGGTGAAGGACACT-3′ for *cspA*; 5′-GCGCACGAAGAGTACGG-3′ for *hns* and 5′-CAGGGGTTTTTGGGTTACCT-3′ for *fis*.

### Electrophoretic mobility shift assay (EMSA)

The 310 bp DNA fragment, excised from pTZ310 by BamHI/HindIII digestion, was end-labeled with α-[^32^P]-dATP by fill-in reaction using the DNA polymerase Klenow fragment. About 5–10 ng of the radioactive DNA fragment were incubated with the indicated amounts of purified FIS and H-NS at 25°C in a reaction mixture (15 μl) containing 10 mM Tris HCl (pH 8), 10 mM MgCl_2_, 100 mM NaCl, 10 mM KCl, 1 mM spermidine, 0.5 mM dithiothreitol, 5% glycerol, 0.08 mg ml ^−1^ BSA, and 50 ng Poly dI-dC as competitor DNA. After 15 min of incubation, samples were subjected to electrophoresis on 6% polyacrylamide gel in TBE buffer (Sambrook and Russell, 2001).

The combined EMSA-Western blot analysis was carried out essentially as described above except that each reaction mix contained 120 ng of a cold DNA fragment corresponding to *cspA* promoter. This fragment (340 bp) was obtained by PCR amplification using the primer pairs 5′-CAACCCGGCATTAAGTAAGC-3′ and 5′-CCATTTTACGATACCAGTCA-3′ on a 1200 bp DNA fragment cloned in pTZ19R (Brandi et al., 1996). Samples were loaded in duplicate on 6% polyacrylamide gel which was subsequently electro-transferred (35 min at 2.5 mA/cmq) into a nitrocellulose membrane. The filter was divided into the two duplicates: one half was incubated with polyclonal antibodies anti-FIS and the other half with antibodies anti-H-NS. Finally, proteins detection was carried out using Alkaline phosphatase Conjugated anti-rabbit IgG and NBT/BCIP as substrates.

### DNase I footprinting

The DNA fragment used in footprints was excised from pTZ310 with BamHI/PstI or HindIII/SmaI and end-labeled by fill-in reaction with α-[^32^P]-dATP using Klenow fragment of DNA polymerase. The radioactive DNA was pre-incubated with the indicated amounts of FIS or/and H-NS for 20 min at 25°C in 30 μl of *in vitro* transcription buffer (see below). After addition of 15 ng of DNase I, the reaction was prolonged for further 45 s and then stopped on ice with 1.5 μl of 0.5 M EDTA (pH 8) and 10 μl of 10 M NH_4_ acetate (pH 7.3). Partially digested DNA was ethanol precipitated in presence of 1 μg of tRNA as carrier and then loaded on a 7% polyacrylamide-urea gel (Sambrook and Russell, 2001).

### *In vitro* transcription

*In vitro* transcription assays were programmed with pKK*cspA310::cat*, a pKK232-8 derivative, carrying a 310 bp DNA fragment of *cspA* promoter region (from pos. −145 to pos. +165; Goldenberg et al., 1997). The reactions, carried out at 37°C in 25 μl of transcription buffer (10 mM Tris HCl, pH 8, 10 mM MgCl_2_, 100 mM NaCl, 2 mM spermidine, 2 mM dithiothreitol, 0.1 mg ml^−1^ BSA), contain 0.15 U of *E. coli* RNA Polymerase (USB), 0.5 mM of each NTP, 5 U of human placental ribonuclease inhibitor and 100 ng of DNA template. At the indicated times, the reaction was stopped with 1.5 μl of 0.5 M EDTA (pH 8) and 10 μl of 10 M NH_4_ acetate (pH 7.5) and mRNA ethanol precipitated. The amount of the *cat* reporter gene transcribed *in vitro* was determined by Northern blotting, probed with a ^32^P-labeled *cat* fragment and quantified by Molecular Imager (Bio-Rad, model FX).

## Results

### The cold-shock *cspA* gene is expressed at 37°C

**Figure 1:**
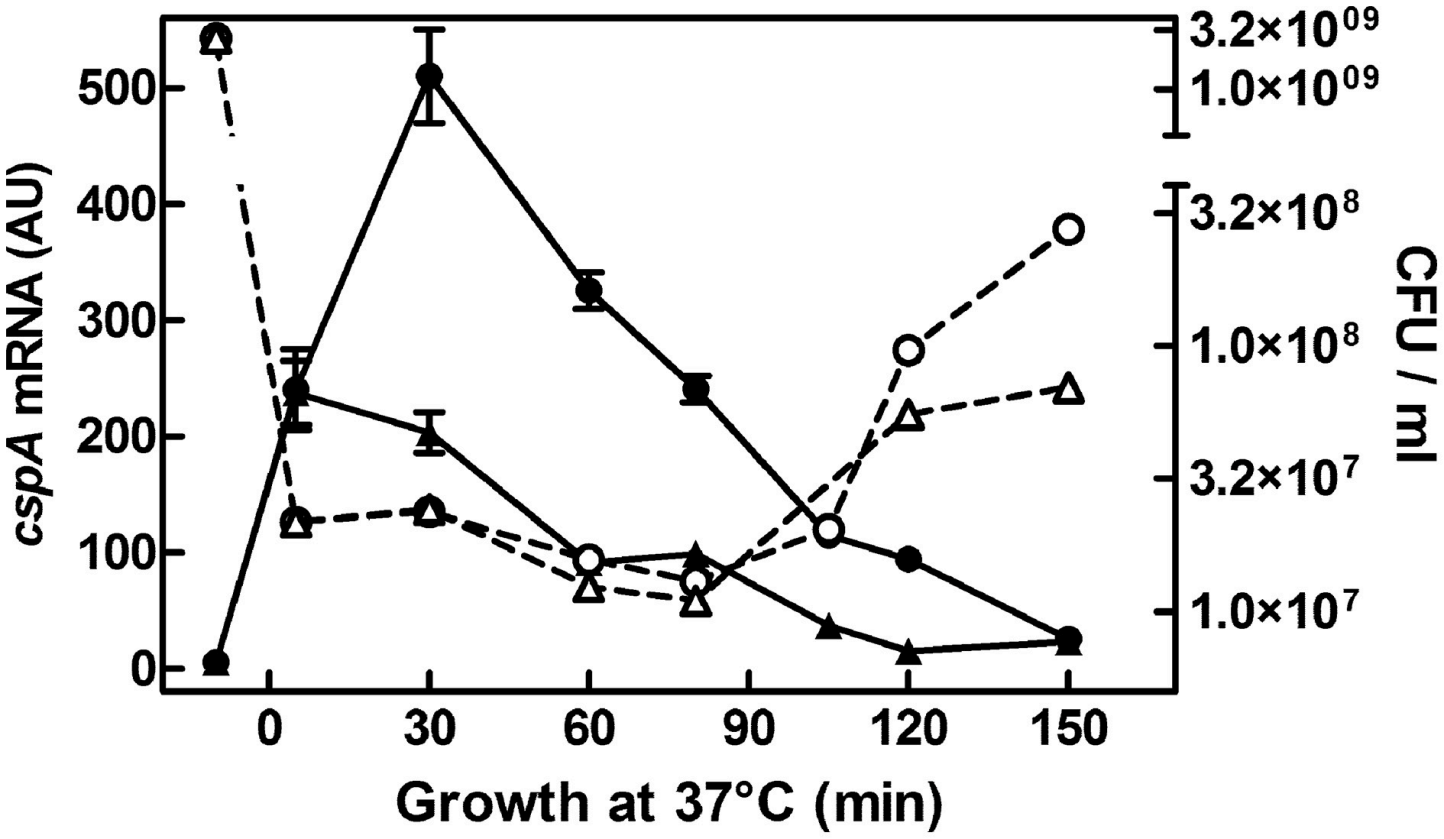
*****cspA*** mRNA steady state level following growth resumption at 37°C**. An overnight culture of *E. coli* MRE600 grown at 37°C in M9 medium was diluted (1:100) in parallel with LB (●) or with M9 (▴) fresh medium and cell aliquots were harvested for RNA extraction at the indicated time after resumption from stationary phase. About 6 μg of total RNA were subjected to Northern analysis. The cellular level of *cspA* transcripts were evaluated by imager quantification of the radioactivity associated with the mRNA and expressed as Arbitrary Units (AU). Data represent the average of at least two independent experiments and standard deviation is indicated. The profile of Colony Forming Units (CFU) during growth in rich (LB,○) or minimal (M9, Δ) medium as a function of time after nutritional up-shift is also reported. Further details are provided in Materials and Methods.

In previous studies, we have shown that *cspA* is highly expressed not only during cold-shock but also under non-stress conditions when cells grow at 37°C (Brandi et al., 1999; Brandi and Pon, 2012). Here, to understand how the physiological state of the cell could affect the expression of *cspA*, we monitored the *cspA* mRNA levels in cells escaping from stationary phase as a function of the availability of nutrients. To accomplish this goal, an overnight culture of *E. coli* grown at 37°C was diluted in rich (LB) or minimal (M9) fresh medium and bulk RNA, extracted from cells at increasing times after the nutritional up-shift, was used for Northern analysis (Figure 1). Transcription of *cspA* is promptly induced upon cell dilution, in both media, albeit to different extents (~40 fold in M9 and ~100 fold in LB). Furthermore, the level of transcript augments within the initial 80 min, a time preceding the first bacterial division as denoted by the constant number of cells (CFU), and drops off to the initial level after the cells start dividing. These observations are confirmed by detecting the *cspA* mRNA by RT-qPCR in cells growing in LB (Figure S6C). In light of this finding, we focused our study on those factors, likely affecting *cspA* regulation at 37°C, that are known to couple gene expression to growth conditions.

### Interaction of H-NS and FIS with the *cspA* promoter region

**Figure 2:**
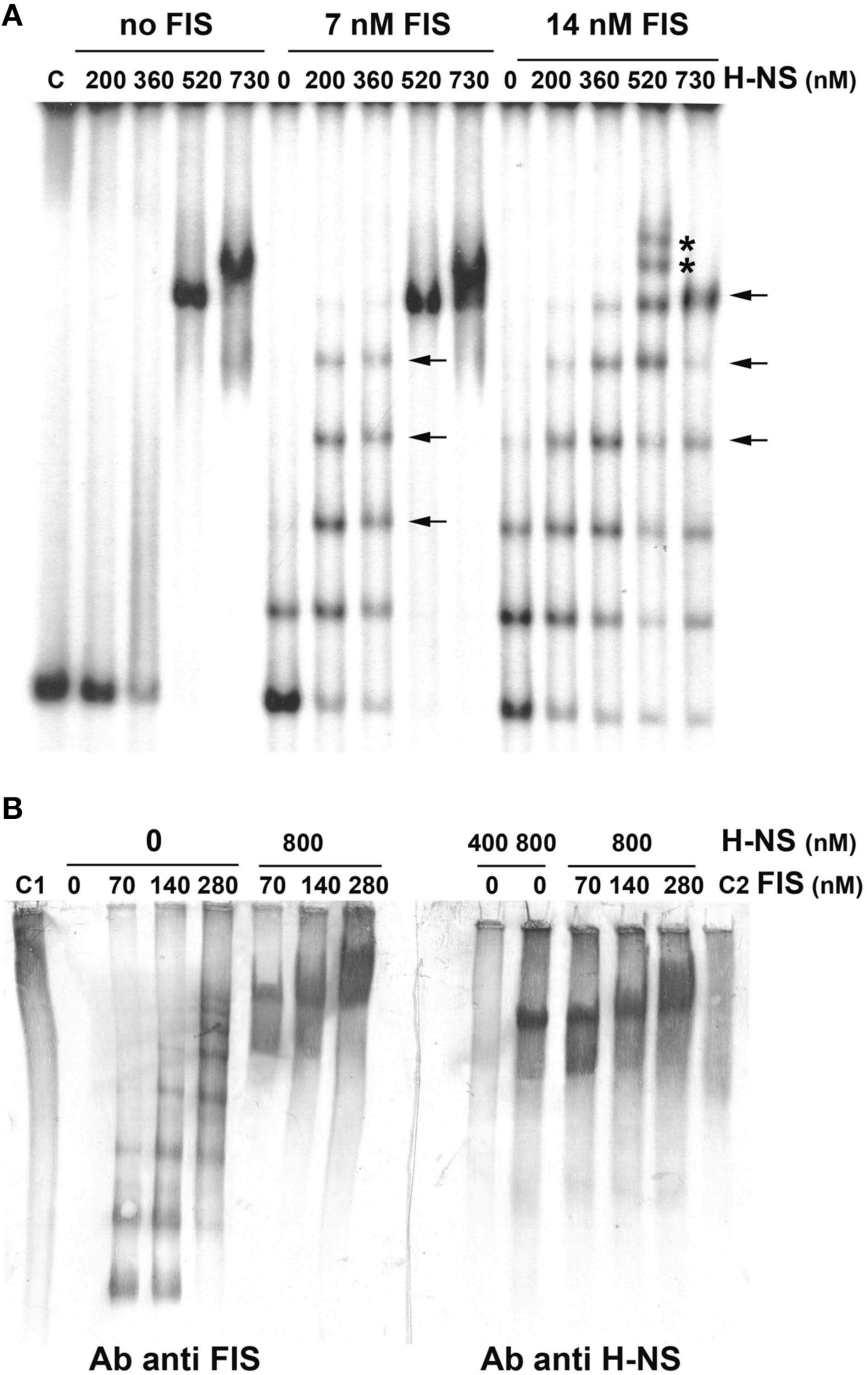
**Band shift of a DNA fragment containing the ***cspA*** promoter by FIS and H-NS**. **(A)** The 310 bp DNA fragment, containing the *cspA* promoter region including the entire 5′-UTR, labeled with ^32^P, was used in electrophoretic mobility shift assay (EMSA) with the indicated amounts of H-NS and/or FIS. DNA-protein complexes formed preferentially or exclusively in the presence of both FIS and H-NS are indicated by arrows or asterisks, respectively. **(B)** EMSA experiments, carried out in duplicate, were subjected to Western Blot. After electrophoresis and elettroblotting, membranes were alternatively developed with antibodies anti-FIS or anti-H-NS as indicated. Lanes C1 and C2 represent the free FIS and H-NS proteins without DNA, respectively. Proteins concentration is given assuming dimeric structure. For further details see Materials and Methods.

Although, FIS and H-NS have been shown to influence *cspA* expression (Brandi et al., 1999), so far the evidence of a direct binding of these proteins to the promoter region of the target gene was lacking. Thus, we investigated the interaction of these two NAPs with the *cspA* DNA by electrophoretic mobility shift assay (EMSA). For this purpose, a fragment of 310 bp spanning from position −145 to position +165, was incubated with H-NS, FIS or with a mixture containing both proteins at different molar ratios. As seen in Figure 2A, when tested individually, H-NS produces an EMSA pattern typical of an all-or-none response, suggesting that this protein binds in a co-operative manner to the 310 bp DNA fragment containing the *cspA* promoter as described for other genetic systems (Falconi et al., 1993, 1998; Madrid et al., 2002; Giangrossi et al., 2005; Ulissi et al., 2014). In the absence of FIS, H-NS has little or no effect below the critical concentration of 360 nM, whereas it forms a stable nucleoprotein complex at 520 nM (Figure 2A). The addition of more H-NS (730 nM) causes the appearance of a new band with reduced mobility, suggesting that, at the maximum concentration tested, H-NS oligomerizes occupying all high and low affinity sites presents on this DNA fragment. Unlike H-NS, a discrete retardation band is detected even at very low FIS concentrations (7 nM) and additional bands with progressively lowered mobility appear as a function of increasing FIS concentrations (14 nM in Figure 2A, 28 and 56 nM in Figure S1). This pattern suggests that the 310 bp DNA fragment contains multiple sites for which FIS displays different affinities and that are saturated in a concentration-dependent manner by this protein. Furthermore, compared to H-NS, a relative little amount of FIS is sufficient to occupy, at least partially, all sites.

Interestingly, the addition of 200–300 nM of H-NS to low concentrations of FIS produces retarded bands (indicated by horizontal arrows in Figure 2A, Figure S1) with a mobility similar to those bands present when FIS alone is added at higher concentrations. Nevertheless, when both H-NS and FIS are added at a certain concentration ratio, additional bands (indicated by asterisks in Figure 2A, Figure S1), not found in the individual H-NS and FIS patterns, appear. On the other hand, when FIS and H-NS are added at low (≤14 nM) and high (>500 nM) concentrations, respectively, the most retarded complex seems to prevalently contain H-NS, since it displays a mobility similar to that obtained with H-NS alone at 520 nM (Figure 2A). All together, these observations indicate that FIS and H-NS might simultaneously bind to the same DNA molecule (the 310 bp DNA fragment) when are present in a given range of concentration ratios.

To verify this hypothesis, we carried out a band shift assay in which the DNA fragment was not radioactive and therefore nucleoprotein complexes were immunodetected using antibodies against FIS or H-NS (Figure 2B). Under the native conditions used for electrophoresis, unbound FIS and H-NS appear diffused throughout the lane (control samples C1 and C2) whereas discrete retarded bands are visible only in the presence of DNA. As expected, when FIS and H-NS are individually tested, DNA-protein complexes visualized by antibodies are superimposable to those obtained with labeled DNA (compare panels B and A of Figure 2). Nevertheless, when FIS (70–280 nM) and H-NS (800 nM) are combined, the same DNA-protein aggregates are revealed using either anti-FIS or anti-H-NS antibodies (Figure 2B). The control experiment shown in Figure S2 rules out the possibility that this result could be due to a cross-reaction between anti-FIS and H-NS or anti-H-NS and FIS. Therefore, all together, these data strongly suggest that both proteins can simultaneously interact with the 310 bp DNA fragment containing the *cspA* promoter to originate miscellaneous complexes.

### Identification of FIS and H-NS binding sites on *cspA* promoter region

**Figure 3:**
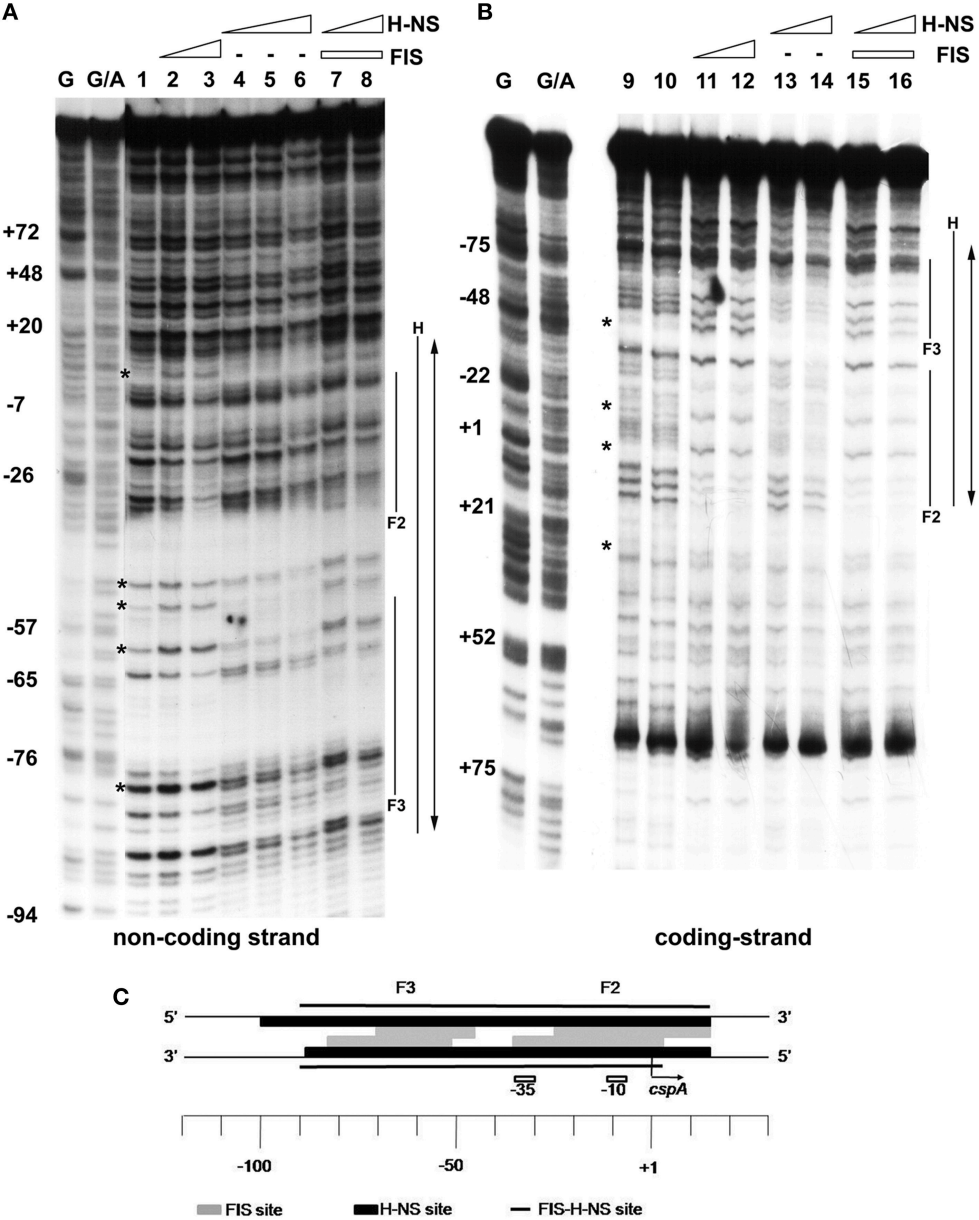
**Mapping of FIS and H-NS binding sites on ***cspA*** promoter region by DNase I footprinting**. Footprinting analysis was carried out on both non-coding strand **(A)** and coding strand **(B)** of a 310 bp DNA fragment corresponding to *cspA* promoter in the presence of the following FIS and HNS dimeric concentrations: no proteins, lanes 1, 9, and 10; 278 nM FIS, lanes 2 and 11; 555 nM FIS, lanes 3 and 12; 93 nM H-NS, lanes 4 and 13; 185 nM H-NS, lanes 5 and 14; 277 nM H-NS, lane 6; 278 nM FIS and 93 nM H-NS, lanes 7 and 15; 278 nM FIS and 185 nM H-NS, lanes 8 and 16. FIS and H-NS sites are indicated by solid lines while the double-headed arrows show protections resulting from the concomitant bond of FIS and H-NS. Sites hypersensitive to DNase I due to FIS-DNA interaction are indicated by asterisk. G and G+A represent the Maxam and Gilbert sequencing reactions. Localization of FIS (gray boxes), H-NS (black boxes), and FIS-H-NS (solid line) binding sites are schematically indicated on *cspA* promoter region **(C)**.

EMSA experiments prompted us to localize FIS and H-NS binding sites on *cspA* promoter, in an attempt to characterize also the nature of the complexes containing both proteins. To this end, we carried out DNase I footprints and compared the digestion patterns obtained with single proteins to that observed with a mixture of FIS and H-NS (Figures 3A,B). When individually tested, FIS interacts with two sites of ~35 bp in length centered at positions −10 (site 2) and −60 (site 3) producing typical hypersensitive points, while H-NS covers a fairly wide region (~100 nucleotides) centered at position −40. When mixed together FIS and H-NS cover all the available sites, giving rise to an extended protection spanning from position −90 to position +20 on *cspA* promoter. Remarkably, the DNAse I digestion pattern observed at this cumulative site is essentially a merge of protected and hypersensitive positions characteristic of FIS and H-NS individual sites. Therefore, although FIS and H-NS protections are almost completely overlapping on both DNA strands (see scheme in Figure 3C), the two proteins apparently do not compete for binding to the same target DNA.

**Figure 4:**
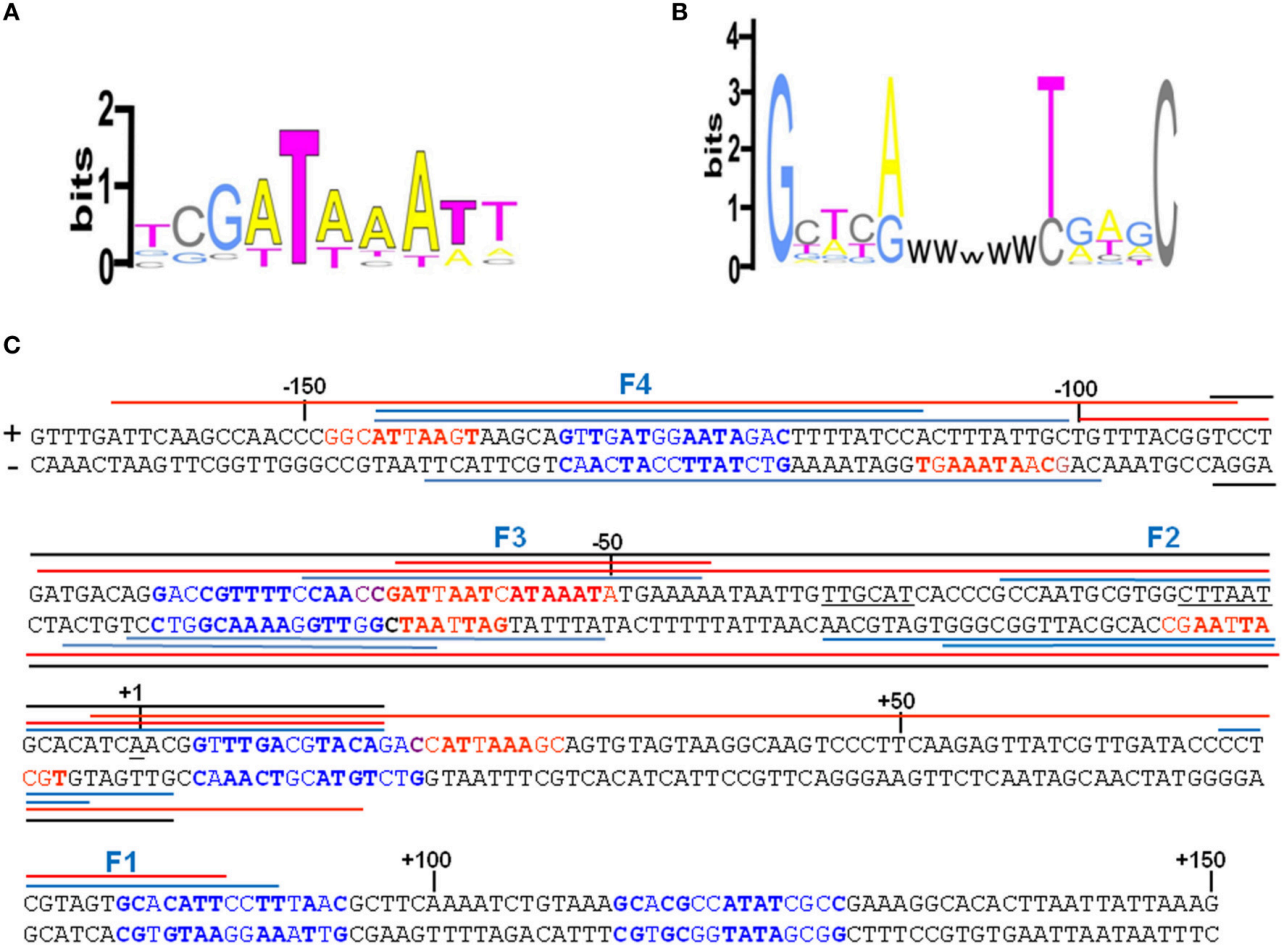
**Localization of FIS and H-NS binding sites found within the ***cspA*** promoter region**. **(A)** Logo representation of the H-NS binding motif (Lang et al., 2007). **(B)** Simplified version of the Logo for the FIS binding motif proposed by Shao et al. (2008). W = A or T. **(C)** The sequence of the promoter region of *cspA* (−167 to +150) is shown, with the transcriptional start point (+1), the −10 and −35 consensus promoter elements underlined in black. The bases in red represent the *in silico* predicted H-NS binding sites matching at least 4/6 bases of the core sequence of the H-NS binding motif and displaying less frequent bases in the other positions, while the bases in blue represent the *in silico* predicted FIS binding sites according to the FIS logo shown in **(B)**. The bases in bold match those found more frequently in the H-NS and FIS binding sites, respectively. The colored lines placed above or below the + and − strand, respectively, indicate the protections from DNase I digestion found with H-NS alone (red), FIS alone (blue), or with a mixture of FIS and H-NS (black). Overlapping lines indicate DNase I footprintings found in independent experiments (Figure 3, Figures S3, S4 in Supplementary Material and other not shown). The protections were identified using either a 310 bp *cspA* fragment (for FIS, H-NS, and FIS+H-NS) or a 1200 bp *cspA* fragment (for FIS and H-NS alone).

An extensive scanning of the regions adjacent to the *cspA* minimum promoter (Figures S3, S4) reveals the existence of other positions recognized by these nucleoid proteins. FIS covers two other distinct sites numbered F1 and F4 and centered at positions +32 and −120, respectively, while H-NS extends its protection to basically the entire *cspA* promoter due to its extensive oligomerization (Spurio et al., 1997; Badaut et al., 2002; Stella et al., 2005; Giangrossi et al., 2014). According to this premise, we found several *in silico* predicted binding sites for H-NS (Figure 4A) that are all imperfect fits to its consensus sequences (Lang et al., 2007) and can be considered as nucleation sites where the protein initially binds before spreading to adjacent DNA tracts on *cspA* sequence. The same *in silico* approach allowed us to identify also five potential FIS binding sites matching the FIS Logo (Figure 4B) proposed by Shao et al. (2008), four of which fall in the regions shielded or exposed to DNase I cleavage by FIS. The overall H-NS and FIS protections on the sequence of *cspA* regulatory region along with their *in silico* predicted binding sites are summarized in Figure 4C.

### Transcription of *cspA* is modulated by FIS, H-NS, and (p)ppGpp

**Figure 5:**
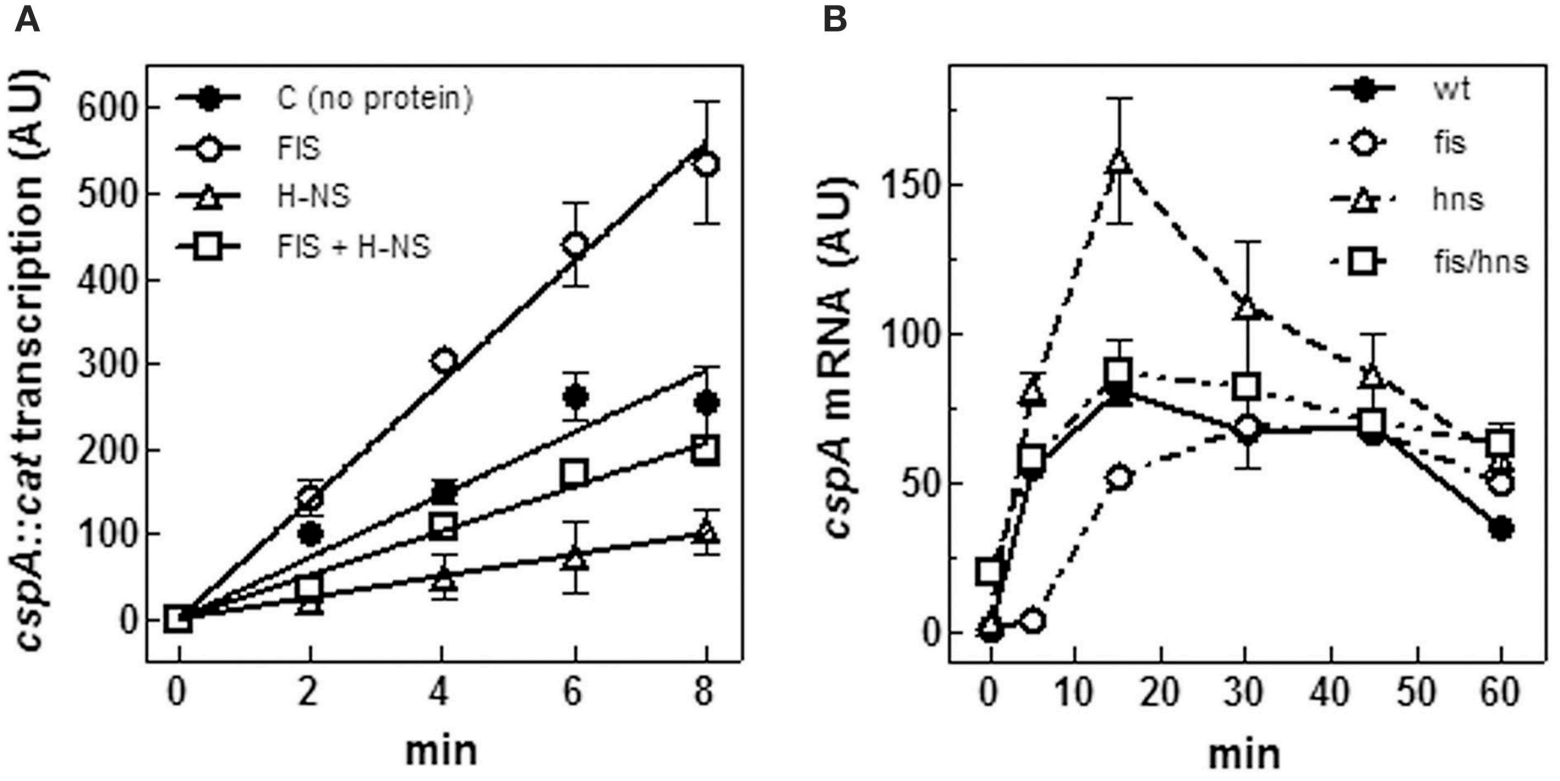
**FIS and H-NS modulate ***cspA*** transcription**. **(A)** *In vitro* transcription assay programmed with pKK*cspA310::cat*. DNA template was pre-incubated at 37°C in the absence of proteins (●), with 50 nM FIS (○), 375 nM H-NS (Δ) and with both FIS (50 nM) and H-NS (375 nM) (□) as dimers. The reaction was started by adding 0.15 units of RNA polymerase and the incubation was prolonged for the indicated times at 37°C, as described in Materials and Methods. **(B)** Steady-state levels of CspA mRNA were determined in wt *E. coli* cells (●), a *fis* null allele (○), an *hns* null allele (Δ), and a double mutant *fis-hns* (□). After a 10-fold dilution with fresh medium of saturated cultures grown in LB at 37°C, total RNA was extracted at the indicated times and subjected (10 μg) to Northern analysis. The radioactivity associated with *cspA* mRNA, normalized for the corresponding amounts of 16S rRNA, was quantified by Molecular Imager (Bio-Rad) and expressed as Arbitrary Units (AU). Data represent the average of at least two independent experiments and standard deviation is indicated.

In a previous paper, we provided evidence of a functional antagonism between FIS and H-NS on *cspA* expression (Brandi et al., 1999). This observation is consistent with the location, reported here, of H-NS and FIS binding sites, extending over the whole promoter region of *cspA*. Concerning the role of these two NAPs, while the inhibitory action of H-NS is commonly accepted, the function of FIS is still a point of debate since contradictory results have been reported about this protein that was also found to negatively regulate *cspA* (Yamanaka and Inouye, 2001). Thus, to address this issue, we assayed H-NS and FIS for their capability to affect *cspA* transcription *in vitro*. As seen in Figure 5A, the activity of *cspA* promoter is stimulated by FIS and repressed by H-NS, totally confirming our previous data. Under the experimental conditions used, the extents of FIS stimulation and H-NS inhibition are similar (~3-fold) as compared to transcription carried out in the absence of proteins. Consistently, when FIS and H-NS are added together, their opposed effects on transcription neutralize each other, thus restoring the basal activity of *cspA* promoter (compare C and FIS+H-NS curves in Figure 5A).

The effect of H-NS and FIS on *cspA* expression was also studied *in vivo*. To this end, the steady state level of *cspA* mRNA was estimated at 37°C upon resumption from stationary phase of growth in wt, *hns*- and *fis*- strains and in *hns*/*fis* double deletion mutant. In agreement with Brandi et al. (1999) and Brandi and Pon (2012) in wt cells *cspA* expression is very high in early exponential growth and then progressively declines (Figure 5B). Interestingly, the lack of FIS causes a reduction of *cspA* transcript as compared to the wt within the initial 15 min, a time window that usually precedes the first cell division. However, at later stages, wt and *fis*- cells show comparable amounts of *cspA* mRNA (Figure 5B). On the contrary, inactivation of *hns* gene induces an increase of the *cspA* mRNA level that almost doubles in the first 20 min of growth with respect to the wt condition, and then declines. Finally, in agreement with the *in vitro* transcription assay (Figure 5A), the concomitant absence of FIS and H-NS results in a compensatory effect and ultimately has no significant consequences on the level of *cspA* mRNA (Figure 5B). Taken together, *in vitro* and *in vivo* data are fully consistent with each other and strongly indicate that H-NS and FIS, acting as negative and positive regulators, respectively, play an opposed role in modulating *cspA* transcription.

The alarmone guanosine tetraphosphate ((p)ppGpp) is a global regulator which is produced in most circumstances and modulates bacterial physiology (Hauryliuk et al., 2015). This small effector molecule is known to influence the expression of many genes thereby coupling the overall level of transcription to growth-rate (Potrykus and Cashel, 2008). Furthermore, overproduction of this unusual nucleotide prior to cold-shock was reported to lower the induction of most cold-shock genes, including *cspA* (Jones et al., 1992a).

**Figure 6:**
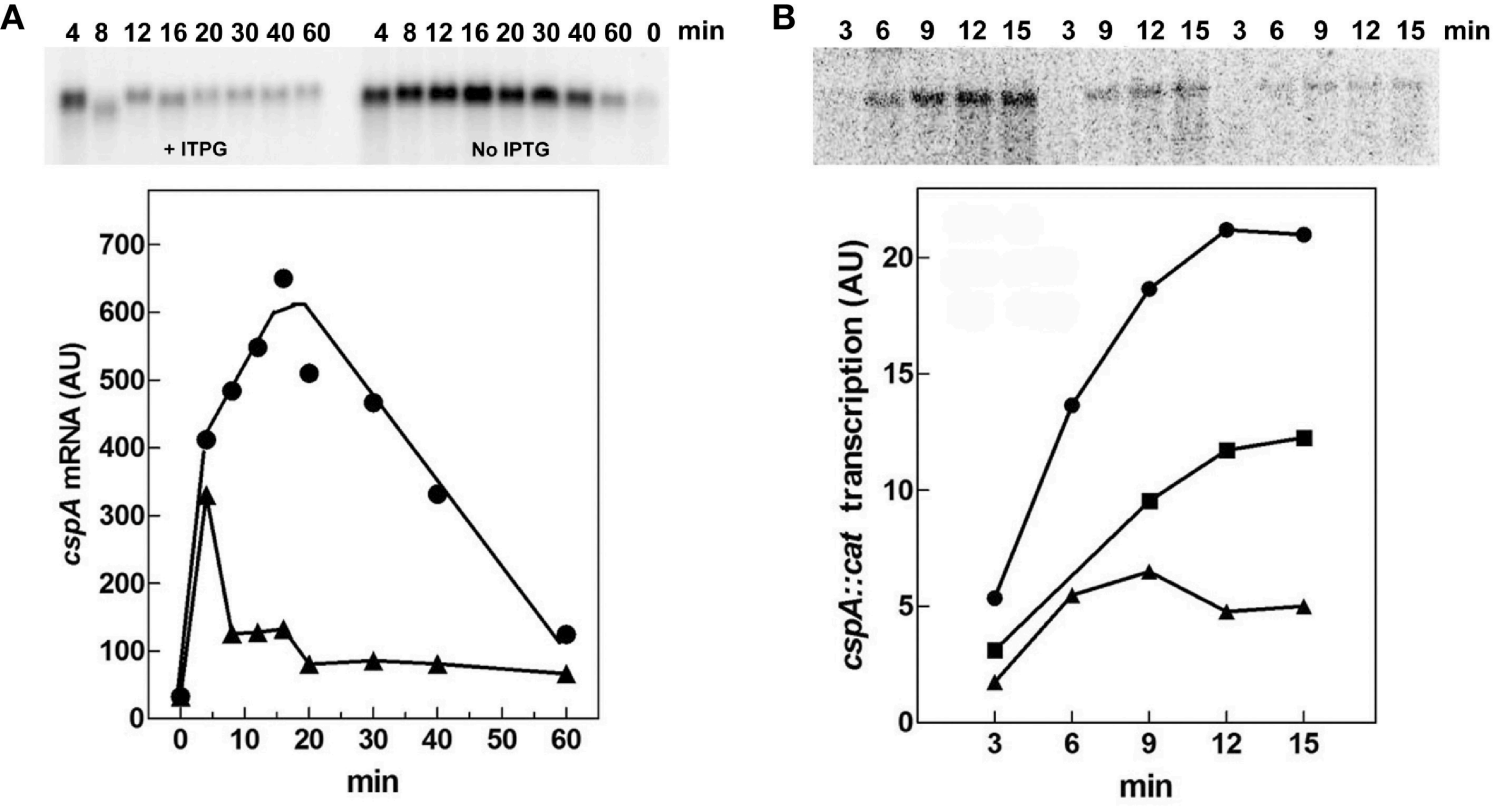
**(p)ppGpp negatively affects ***cspA*** transcription**. **(A)** The steady state levels of *cspA* transcript were determined during growth in Phosphates-free medium at 37°C in cells overproducing or not (p)ppGpp. Total RNA was extracted from cells in stationary phase (time zero) and at the indicated times after dilution with fresh medium alone (●) or supplemented with 400 μM IPTG (▴). The levels of *cspA* mRNA were estimated by Northern blotting analysis. **(B)** Effect of (p)ppGpp on the *in vitro* activity of *cspA* promoter. The transcriptional activity was estimated in the absence (●) or in presence of 200 (■) and 400 μM (▴) of (p)ppGpp. Reactions and processing of samples were performed as described in Materials and Methods. The supercoiled plasmid pKK310::*cat* used as DNA template was pre-incubated with (p)ppGpp and RNA polymerase for 5 min at 37°C. Then reactions were started by adding NTPs at a final concentration of 100 μM each. At the indicated incubation times, aliquots were withdrawn and transcription stopped with EDTA (f.c. 30 mM). Quantization of the Northern blots (upper panels), expressed as Arbitrary Units (AU), is plotted as a function of time (lower panels).

**Figure 7:**
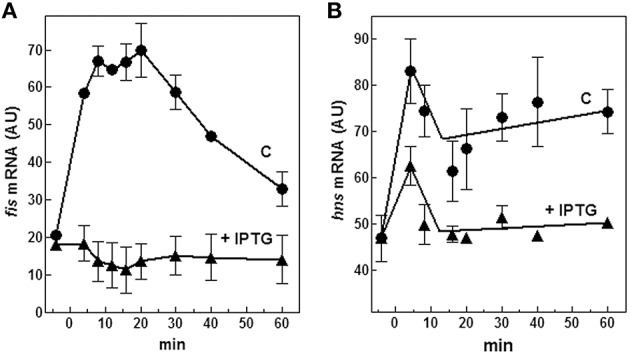
**Effect of (p)ppGpp on ***fis*** and ***hns*** expression**. Levels of *fis* **(A)** and *hns* **(B)** mRNAs were estimated by Northern blotting analysis of total RNA extracted from *E. coli* cells growing at 37°C and carrying the plasmid pTK31. Cells were collected in stationary phase (time zero) and at the indicated times after dilution with fresh medium. As described in Figure 6A, the control culture was grown in the absence of IPTG (▴) whereas the induced culture contained 400 μM IPTG (●) to activate the *lacUV5::relA* gene. Data represent the average of at least two independent experiments and standard deviation is indicated.

Therefore, we evaluated both *in vivo* and *in vitro* whether *cspA* promoter could respond to (p)ppGpp regulation also at 37°C (Figure 6). First of all, the intracellular (p)ppGpp level was artificially increased by IPTG induction of extra-chromosomal copies of *relA* gene placed under the control of the *lacUV5* promoter in plasmid pTK31 (see Materials and Methods). The induction of (p)ppGpp synthesis from pTK31 was verified (not shown) by thin layer chromatography as previously described (Sarubbi et al., 1989). As expected, when cells in stationary phase were subjected to a nutritional up-shift, a sudden burst of *cspA* transcription was observed. By contrast, when the fresh medium was supplemented with IPTG, the high levels of the unusual nucleotide in induced cells significantly counteracted the characteristic promoter activation resulting in a substantial reduction of *cspA* messenger (Figure 6A). Thus, hypothesizing a direct action of (p)ppGpp, we investigated the effect of this molecule on *cspA* promoter activity in an *in vitro* purified system, following *cspA* transcription as a function of increasing reaction times. This experiment demonstrates that the level of *cspA* mRNA is decreased 2- and 4-fold in the presence of 200 and 400 μM of (p)ppGpp, respectively, as compared to the control curve obtained in the absence of the regulatory nucleotide (Figure 6B). According to the finding that (p)ppGpp-dependent inhibition of transcription of sensitive promoters results from the competition between the mediator molecule and NTPs substrates for access to the active center of RNA polymerase (Jöres and Wagner, 2003), the use of higher concentrations of NTPs (0.5 mM) alleviates the negative action of (p)ppGpp on *cspA* transcription (Figure S5). Altogether these results indicate that the stimulation of *cspA* expression, observed in early exponential growth at 37°C, is almost completely prevented by high levels of (p)ppGpp and that this effect reasonably resides on the ability of this molecule to directly repress the synthesis of mRNA from *cspA* promoter. Finally, to better understand the three components (FIS, H-NS, and (p)ppGpp) regulatory loop governing *cspA* expression, we analyzed, under our experimental conditions (cells carrying the pTK31 vector) *fis* and *hns* transcription as a function of increased intracellular concentrations of (p)ppGpp. As seen in Figure 7, activation of *fis* and *hns* promoters consequent to cell resumption from stationary phase is completely (panel A) and partially (panel B) abolished, respectively, by an elevated level of (p)ppGpp suggesting that transcription of both genes is negatively regulated by this alarmone.

## Discussion

There is evidence that regulation of many bacterial genes is based on structural/functional interplays between two or more nucleoid-associated proteins which may play synergetic and/or antagonistic roles. In particular, FIS and H-NS cooperate to regulate several unrelated genetic systems in different bacteria. Some examples are *rrnB* (Afflerbach et al., 1999), *oriC* (Roth et al., 1994), *nir* (Browing et al., 2000), *dps* (Grainger et al., 2008), *pel* (Ouafa et al., 2012) as well as those systems that we have contributed to characterize, such as the FIS-H-NS-mediated regulation of the *E. coli hns* gene itself (Falconi et al., 1996), or the virulence gene *virF* of *Shigella flexneri* (Falconi et al., 2001).

In this study, in addition to confirming our earlier observations on the involvement of FIS and H-NS in controlling *cspA* (Brandi et al., 1999), we dissected their interplay at *cspA* promoter. Furthermore, we identified another factor, the (p)ppGpp, participating in this regulation, thus expanding our knowledge of the regulatory circuit governing the expression of *cspA* at 37°C.

Firstly, through both EMSA and footprinting assays, we have demonstrated the direct interaction of FIS and H-NS with the promoter region of *cspA* and identified their target sites as summarized in Figure 4. The appearance of discrete bands with shifted mobility indicates that FIS binds at least five sites on the *cspA* promoter region spanning from position −167 to position +150. This interaction is also confirmed by the FIS-dependent protections from DNase I cleavage of four discrete stretches of nucleotides (~30–40 bp). Notably, all these protected targets overlap a degenerated FIS consensus sequence predicted *in silico* using a software that allows identification of sequence motifs (Hu et al., 2003) based on the FIS binding site logo (Shao et al., 2008). With regard to H-NS, we found six sequences on *cspA* DNA partially matching the H-NS binding motif as identified by Lang et al. (2007). These sequences may represent high-affinity nucleation sites where H-NS initially binds before oligomerizating along DNA and interacting with adjacent lower affinity sites. Such binding property accounts for the extended regions protected by H-NS in footprinting experiments (Figure 3, Figure S4) and ultimately determines the complete coverage of *cspA* promoter region including the four mapped FIS sites (F1–F4). Both the standard EMSA and a modified EMSA, in which the DNA-protein complexes were localized by immunodetection (Figure 2), reveal that FIS and H-NS, instead of structurally competing for the binding to the DNA, at certain concentration ratios can contact simultaneously the region containing the *cspA* promoter each one recognizing their own targets. The formation of high-order aggregates containing both proteins is also evidenced by the occurrence of a single merged protection observed in footprints carried out with FIS and H-NS together. The coexistence of these two proteins is likely due to their different binding properties: while FIS interacts with the major groove (Osuna et al., 1991), H-NS contacts the minor groove of the DNA, as demonstrated by the finding that two DNA minor groove-binding molecules, distamycin and netropsin, effectively compete with H-NS for the binding to an AT-rich sequence (Yamada et al., 1990; Gordon et al., 2011). Furthermore, our EMSA experiments strongly suggest that the binding affinity of FIS and H-NS for *cspA* DNA increases when the proteins are combined (Figure 2, Figure S1). Two not mutually exclusive circumstances can explain this observation: FIS can bend DNA upon binding (Pan et al., 1996), thus facilitating the interaction of H-NS that is known to preferentially recognize curved DNA (Yamada et al., 1990) or H-NS can actively curve not curved DNA (Spurio et al., 1997), thus easing the positioning of FIS at its binding sites. Since the regions protected by FIS (site F2) and H-NS overlap the −35 and −10 elements of the promoter (Figure 4), their occupancy may allow the formation of contacts between the two nucleoid proteins and RNA polymerase thereby accounting for the stimulation/repression of *cspA* transcription by FIS and H-NS (Figure 5). This scenario shares many similarities with the models proposed for the regulation of *hns* and *virF* genes by H-NS and FIS in which the protein molar ratio reflects the nature of the hetero-complex formed and ultimately controls the switch between a transcriptionally active or repressed state (Falconi et al., 1996, 2001).

The composition of the population of NAPs in the cell is not fixed and fluctuations of these proteins is thought to mediate global changes in nucleoid structure and transcriptional activity (Azam and Ishihama, 1999). In fact, growth phase and variations of other environmental parameters (i.e., temperature, pH, availability of nutrients and oxygen) produce a characteristic profile of nucleoid-associated proteins. While the intracellular level of H-NS is generally high and quite constant, the expression of FIS is strongly dependent on growth conditions, being elevated in early exponential phase and upon a nutrient up-shift (Ball et al., 1992; Dillon and Dorman, 2010).

Interestingly, the expression pattern of *cspA* at 37°C seems to mirror that of FIS, since *cspA* transcription is maximal before growth resumption from stationary phase (in the period of time immediately preceding the first cell division), and it is progressively reduced at later stages of growth. Since FIS is able to directly stimulate the transcription of *cspA* and to contrast the inhibitory effect of H-NS, as demonstrated by *in vitro* transcription assays (Figure 5A), it is likely that changes of FIS intracellular levels help to link the physiological state of cells and the environmental conditions to *cspA* expression. This model of regulation is confirmed also by *in vivo* experiments performed in different genetic backgrounds. In fact, with respect to wt strain, the raise in *cspA* mRNA level during the initial minutes after escape from stationary phase is reduced and delayed in *fis*- background and increased in *hns*- strain (Figure 5B). Furthermore, in line with our model, at increased culture age the *cspA* mRNA level in both wt and *hns*- strains becomes comparable to that of *fis*- strain. Therefore, FIS seems able to bind *cspA* promoter and sponsor the activation of *cspA* transcription in a dose-dependent manner so that when its level drops below a certain concentration, it can no longer contrast the inhibition by H-NS.

In addition to NAPs, *cspA* promoter is able to respond to changes of (p)ppGpp. Although, the intracellular concentration of this mediator molecule is quite stable under physiological growth conditions, its synthesis is affected by several types of nutritional limitations and other environmental stimuli, like an abrupt change of temperature (Magnusson et al., 2005). Here, we show that a high level of (p)ppGpp abolishes the *in vivo* induction of *cspA* after a nutritional up-shift and that this effect is due to a direct inhibition of *cspA* promoter activity (Figure 6). Numerous hypotheses have been made to explain the negative regulation of transcription by (p)ppGpp. These mechanisms, not mutually exclusive but possibly working in concert, rely on three main conditions: (i) the presence of particular features of promoters sensitive to (p)ppGpp; (ii) the direct interaction between (p)ppGpp and RNAP; (iii) the regulatory role of DksA protein (reviewed in Magnusson et al., 2005; Potrykus and Cashel, 2008; Hauryliuk et al., 2015). Interestingly, the *cspA* promoter contains, in the region between the TATA box (−10) and the transcriptional start site (+1), a GC-rich sequence termed discriminator (Figure 4), that is a key element of those promoters repressed by (p)ppGpp. Furthermore, in our genetic system, a direct interaction of (p)ppGpp with RNAP is supported by the fact that the *in vitro* inhibition of *cspA* transcription by this effector is dependent on the amount of NTPs added. In fact, an excess of NTPs (from 0.1 to 0.5 mM) causes a general decreased capability of the alarmone to negatively affect the promoter activity of *cspA* and makes the transcription repression independent from the concentration of (p)ppGpp (compare mRNA levels at 200 and 400 μM of (p)ppGpp in Figure 6B, Figure S5). Similarly, a sensitivity to the nature and concentration of initiating nucleotide was observed in the (p)ppGpp-mediated regulation of rRNA transcription (Jöres and Wagner, 2003; Kolmsee et al., 2011) where the major step affected by (p)ppGpp is the formation of the ternary transcription initiation complex. This finding strongly indicates that the unusual nucleotide and NTPs compete for access to the active center of RNAP and is consistent also with the (p)ppGpp-RNAP cocrystals that positioned (p)ppGpp in the secondary channel of the enzyme near the catalytic center (Artsimovitch et al., 2004). Notably, the amount of (p)ppGpp added in our *in vitro* transcription assays is very close to that used in similar studies (Heinemann and Wagner, 1997) and compatible with that estimated *in vivo* (~900 μM) in response to amino acid starvation (Traxler et al., 2008).

The *cspA* and *fis* genes are apparently regulated in parallel and this may be attributed to the role of the common regulatory molecule, (p)ppGpp. As *cspA*, also *fis* expression is controlled by this unusual nucleotide as demonstrated by the fact that the stringent response produced by the artificial induction of (p)ppGpp in early log cells causes a dramatic reduction of *fis* mRNA while transcription of *hns*, under the same experimental conditions, is less affected (Figure 7). Interestingly, also *fis* promoter contains a GC-rich discriminator sequence downstream the −10 position and its transcription peaks during the early logarithmic growth phase, a condition characterized by a low concentration of (p)ppGpp, to decrease soon thereafter, as the level of (p)ppGpp begins to rise in cells approaching the stationary phase (Mallik et al., 2004). Therefore, (p)ppGpp is a fundamental element of this regulatory circuit and is able to repress *cspA* transcription by a dual mechanism: one direct, exerting a negative action on the functionality of *cspA* promoter and one indirect, depriving the target gene of its natural transcriptional activator FIS. On the other hand, a low level of FIS favors the silencing of *cspA* by H-NS.

A recent expression analysis of *csp* genes based on quantitative RT-PCR (Czapski and Trun, 2014) has demonstrated that the levels of *csp* mRNAs change with growth phase and type of medium. In particular, in rich defined medium the transcripts of *cspA, B, G*, and *I* were found to accumulate preferentially in early log phase, those of *cspC* and *cspD* in mid-log phase and stationary phase, respectively, and *cspE* mRNA was found to be constitutively present. Our comparison of *cspB, G*, and *I* expression pattern using both Northern blotting and quantitative RT-qPCR (Figure S6) essentially confirms that these genes display a growth-dependent expression similar to that of *cspA*. Therefore, it is tempting to speculate that at 37°C also the fluctuations of other *csp* genes could be regulated by a network similar to that found for *cspA* and based on the antagonistic role of FIS/H-NS and the transcription inhibition by (p) ppGpp.

## Author contributions

AB designed and performed most of the experiments giving an important contribution also to the analysis and interpretation of data. Additionally AB in collaboration with MG has been dealing with the preparation of figures, material and methods and references. MG and MF planned and carried out some experiments. MF and AG have mainly done the analysis and interpretation of results, drafting the work (including figures) and revising it critically.

## Funding

Fondi di Ricerca di Ateneo (FAR), Università di Camerino, to MF.

### Conflict of interest statement

The authors declare that the research was conducted in the absence of any commercial or financial relationships that could be construed as a potential conflict of interest.
