# Supplementary material for: An Interplay among FIS, H-NS, and Guanosine Tetraphosphate Modulates Transcription of the Escherichia coli cspA Gene under Physiological Growth Conditions
Source: Front Mol Biosci. 2016 May 24;3:19. doi: 10.3389/fmolb.2016.00019 (PMC4877382; doi:10.3389/fmolb.2016.00019)

## Slide 1
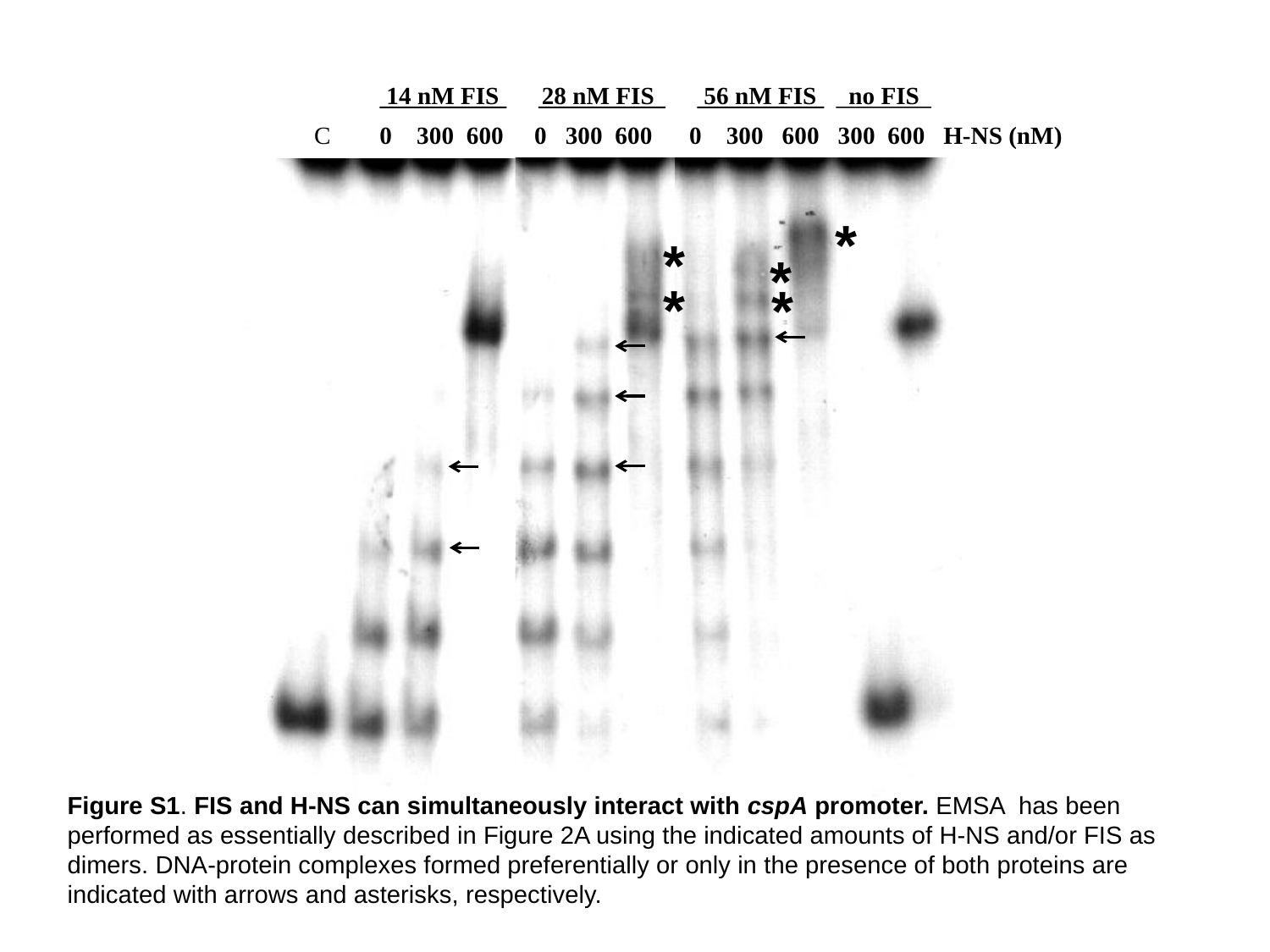

14 nM FIS
28 nM FIS
56 nM FIS
no FIS
C 0 300 600 0 300 600 0 300 600 300 600 H-NS (nM)
*
*
*
*
*
Figure S1. FIS and H-NS can simultaneously interact with cspA promoter. EMSA has been performed as essentially described in Figure 2A using the indicated amounts of H-NS and/or FIS as dimers. DNA-protein complexes formed preferentially or only in the presence of both proteins are indicated with arrows and asterisks, respectively.

## Slide 2
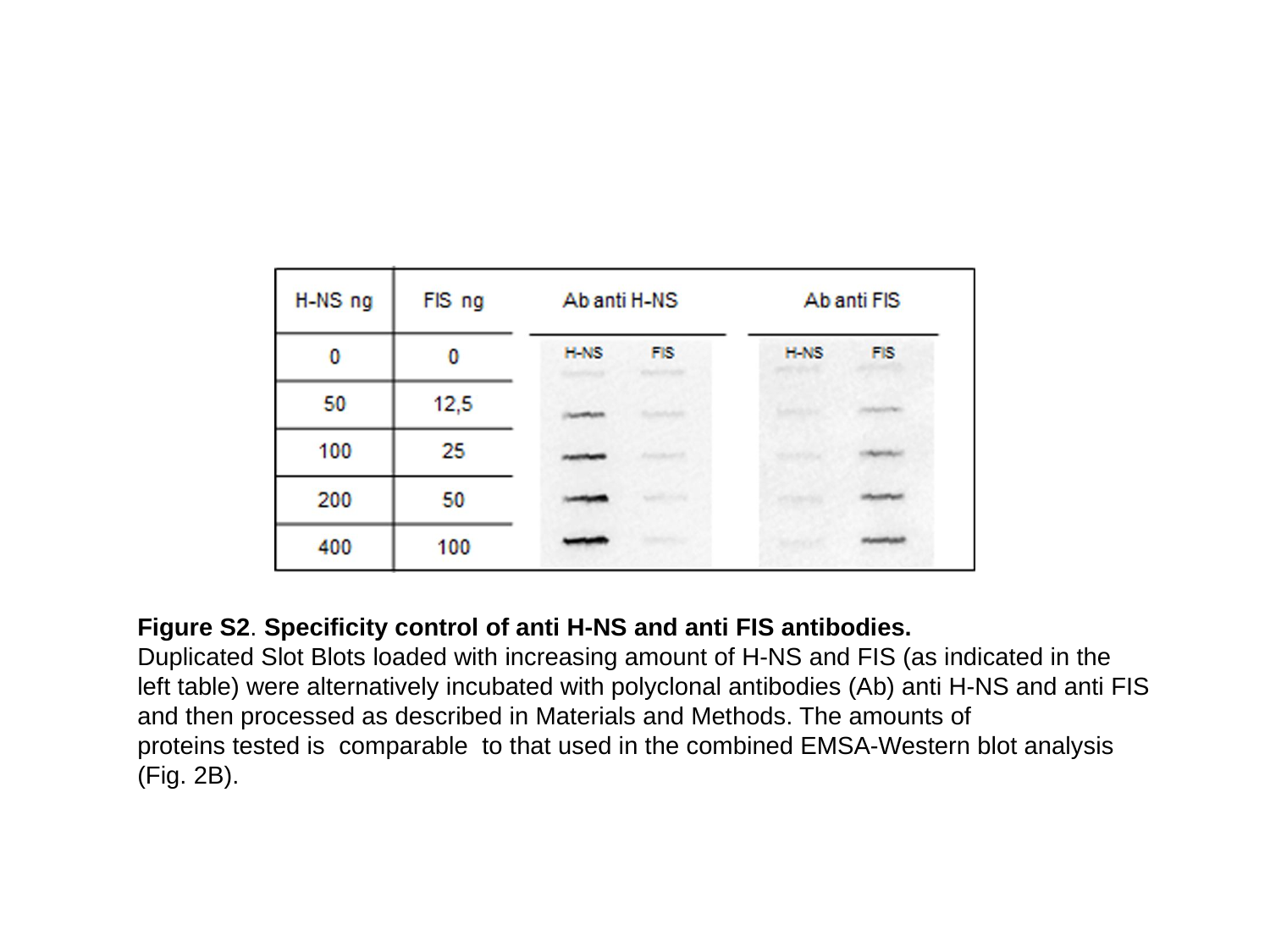

Figure S2. Specificity control of anti H-NS and anti FIS antibodies.
Duplicated Slot Blots loaded with increasing amount of H-NS and FIS (as indicated in the left table) were alternatively incubated with polyclonal antibodies (Ab) anti H-NS and anti FIS and then processed as described in Materials and Methods. The amounts of proteins tested is comparable to that used in the combined EMSA-Western blot analysis (Fig. 2B).

## Slide 3
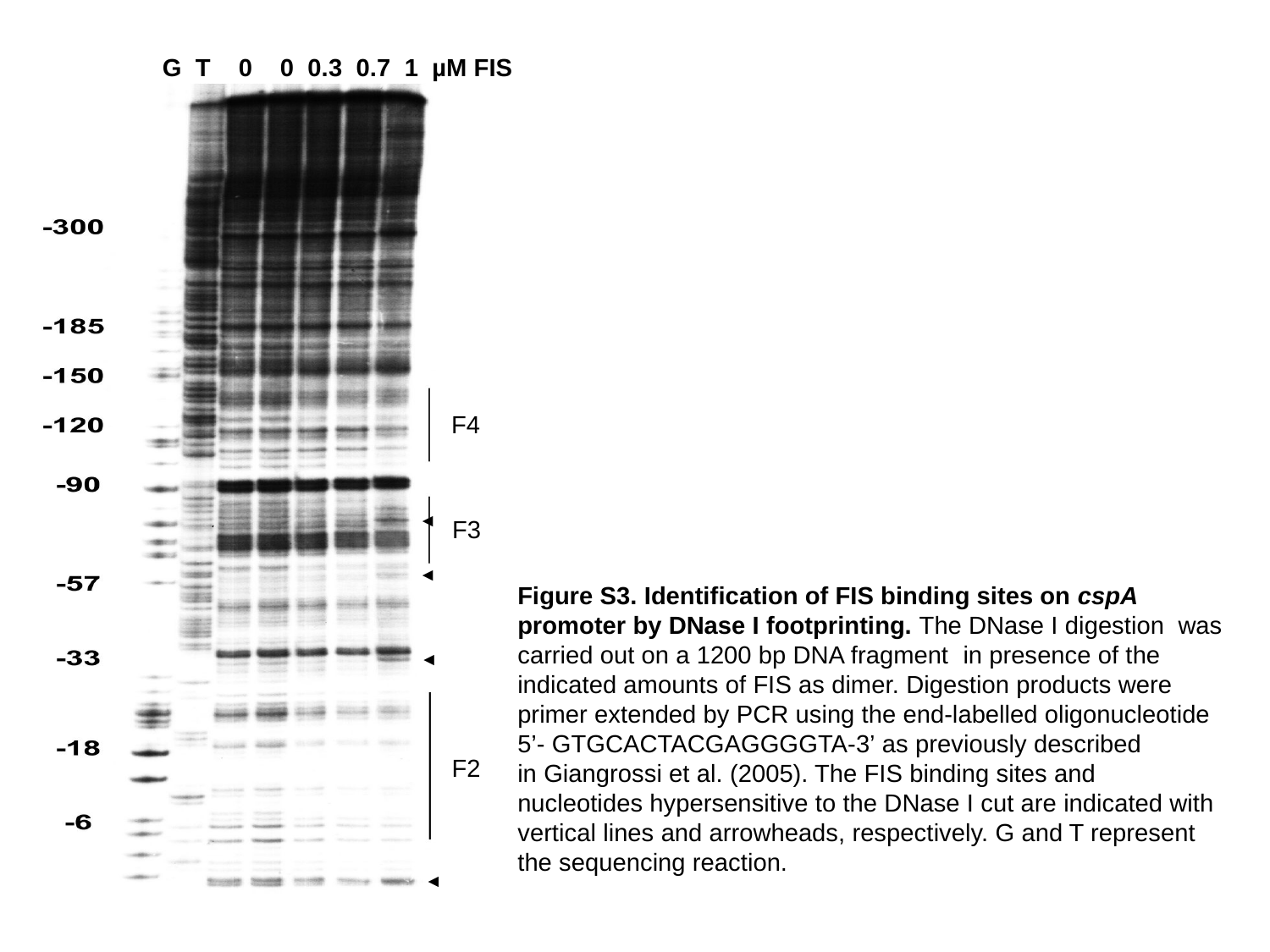

G T 0 0 0.3 0.7 1 µM FIS
F4
◄
F3
◄
Figure S3. Identification of FIS binding sites on cspA promoter by DNase I footprinting. The DNase I digestion  was carried out on a 1200 bp DNA fragment  in presence of the indicated amounts of FIS as dimer. Digestion products were primer extended by PCR using the end-labelled oligonucleotide 5’- GTGCACTACGAGGGGTA-3’ as previously described in Giangrossi et al. (2005). The FIS binding sites and nucleotides hypersensitive to the DNase I cut are indicated with vertical lines and arrowheads, respectively. G and T represent the sequencing reaction.
◄
F2
◄

## Slide 4
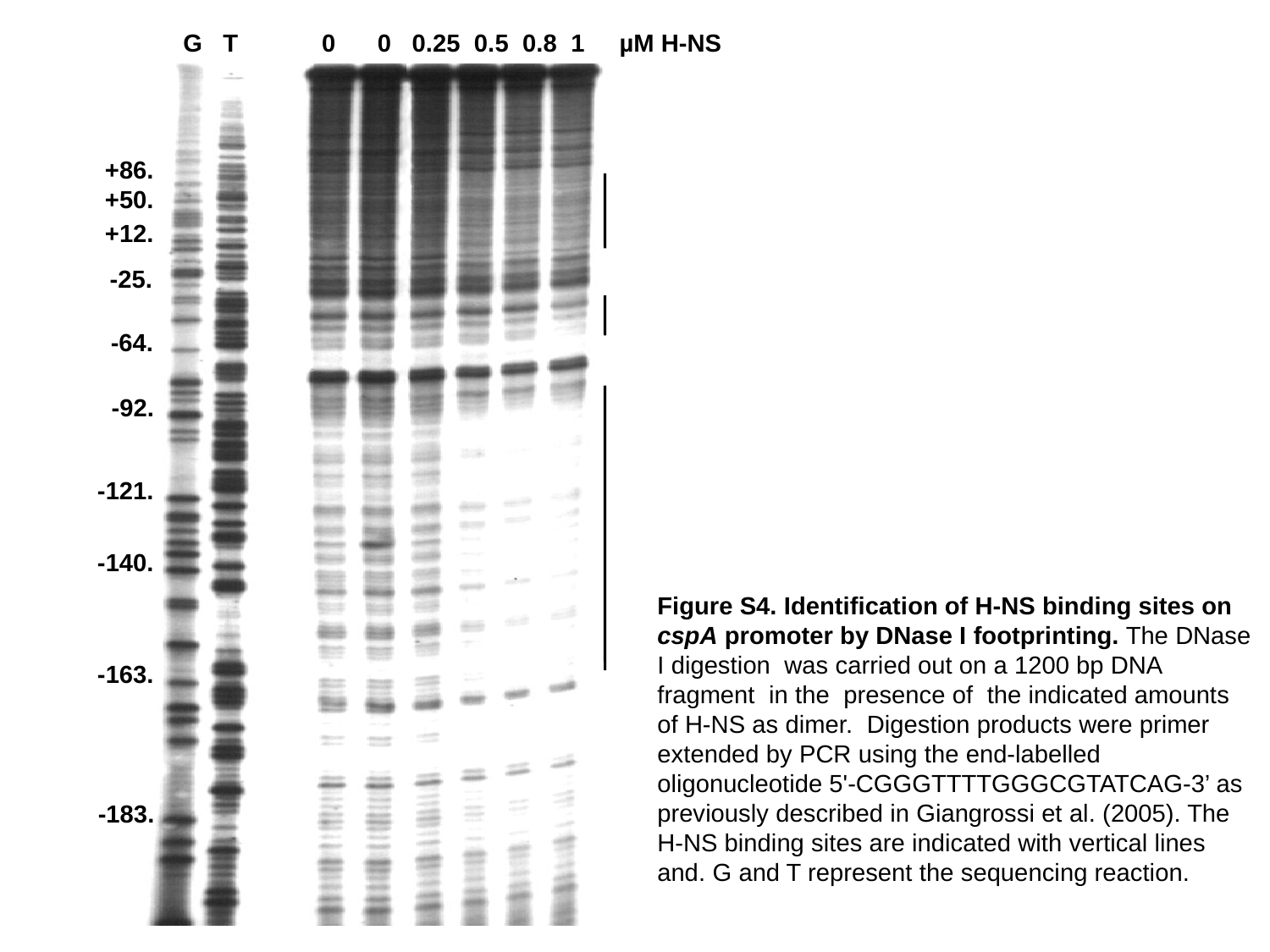

G T 0 0 0.25 0.5 0.8 1 µM H-NS
+86.
+50.
+12.
-25.
-64.
-92.
-121.
-140.
Figure S4. Identification of H-NS binding sites on cspA promoter by DNase I footprinting. The DNase I digestion was carried out on a 1200 bp DNA fragment in the presence of the indicated amounts of H-NS as dimer. Digestion products were primer extended by PCR using the end-labelled oligonucleotide 5'-CGGGTTTTGGGCGTATCAG-3’ as previously described in Giangrossi et al. (2005). The H-NS binding sites are indicated with vertical lines and. G and T represent the sequencing reaction.
-163.
-183.

## Slide 5
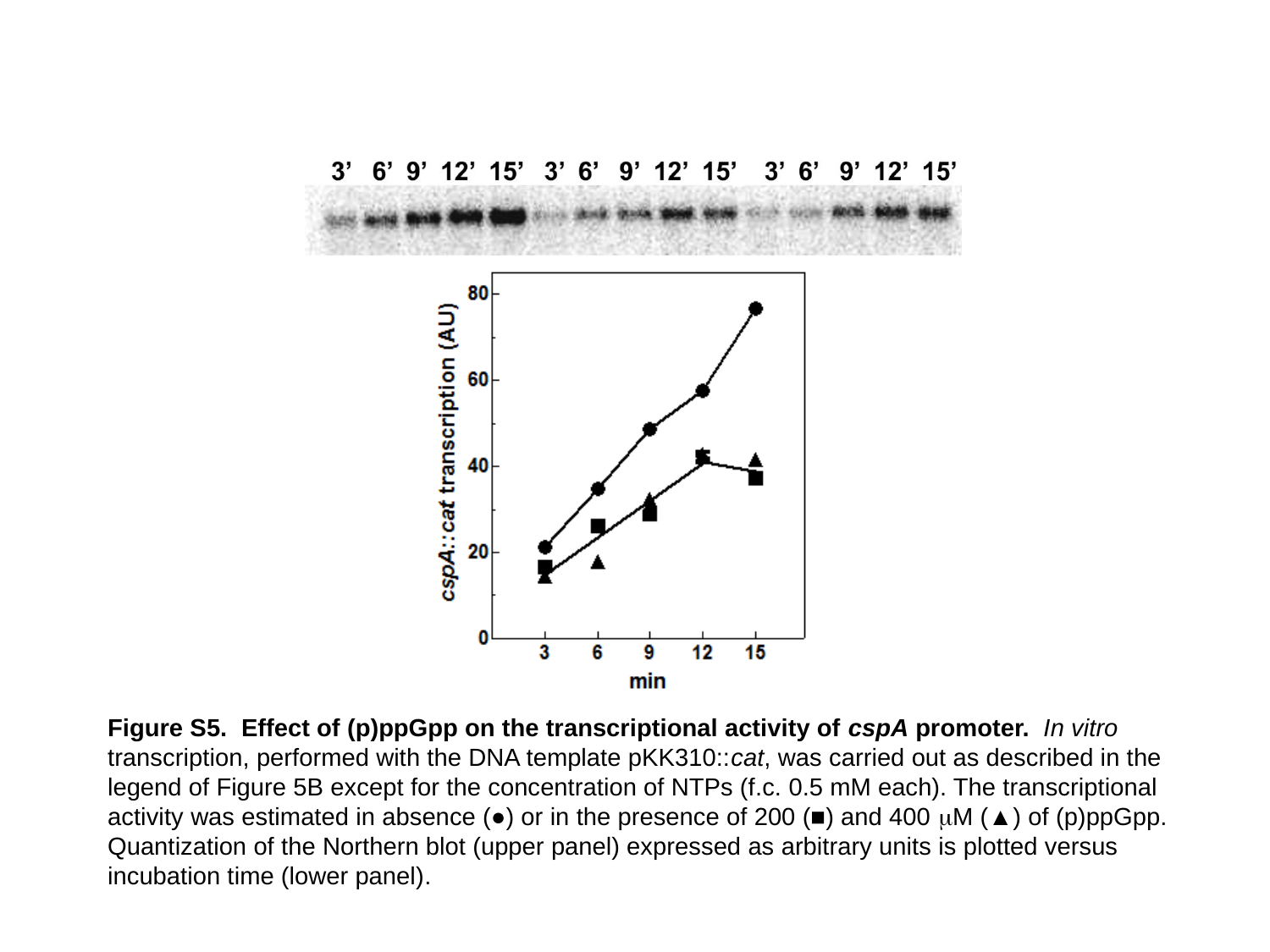

Figure S5.  Effect of (p)ppGpp on the transcriptional activity of cspA promoter.  In vitro transcription, performed with the DNA template pKK310::cat, was carried out as described in the legend of Figure 5B except for the concentration of NTPs (f.c. 0.5 mM each). The transcriptional activity was estimated in absence (●) or in the presence of 200 (■) and 400 mM (▲) of (p)ppGpp. Quantization of the Northern blot (upper panel) expressed as arbitrary units is plotted versus incubation time (lower panel).

## Slide 6
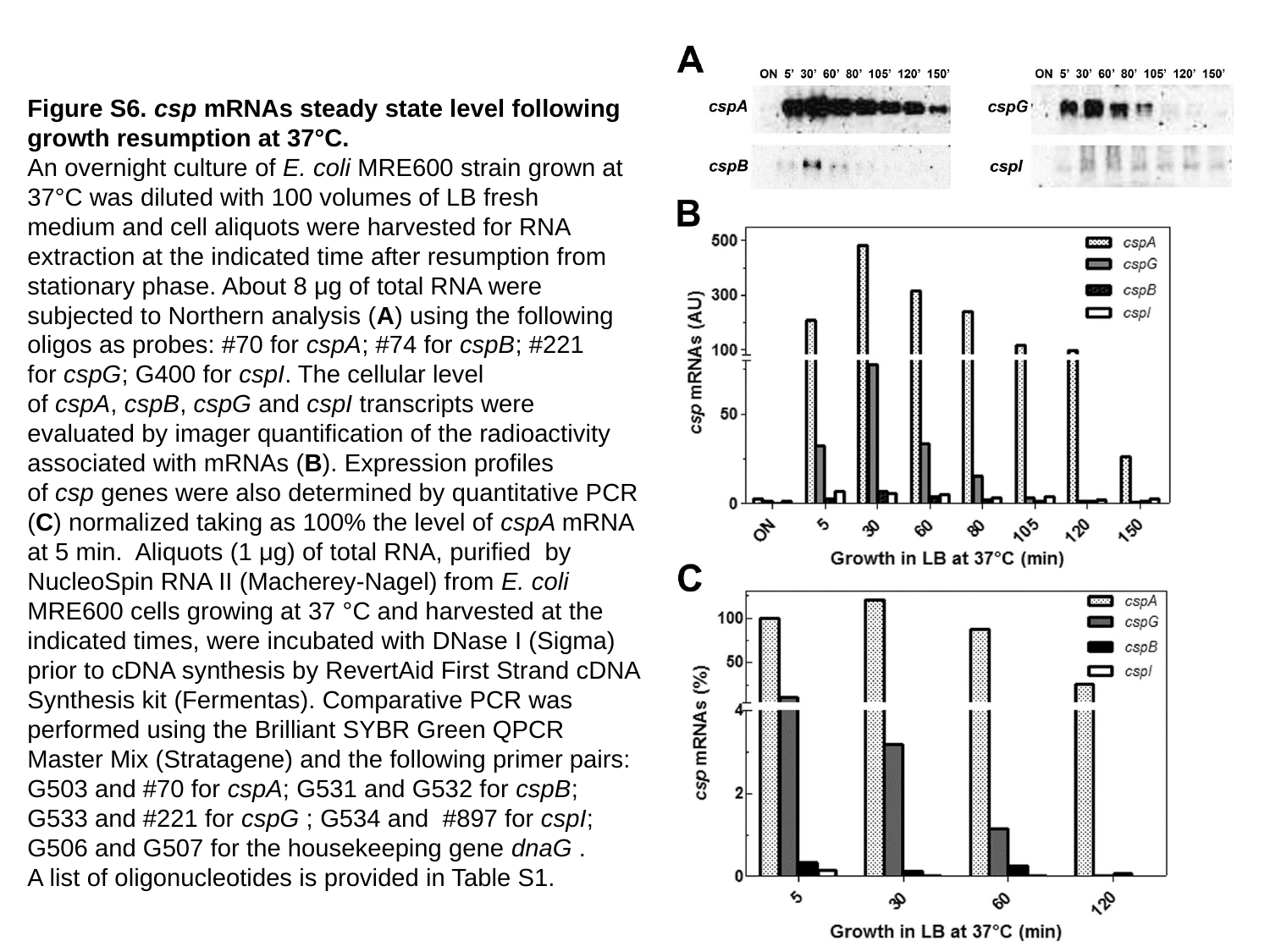

Figure S6. csp mRNAs steady state level following growth resumption at 37°C.
An overnight culture of E. coli MRE600 strain grown at 37°C was diluted with 100 volumes of LB fresh medium and cell aliquots were harvested for RNA extraction at the indicated time after resumption from stationary phase. About 8 μg of total RNA were subjected to Northern analysis (A) using the following oligos as probes: #70 for cspA; #74 for cspB; #221 for cspG; G400 for cspI. The cellular level of cspA, cspB, cspG and cspI transcripts were evaluated by imager quantification of the radioactivity associated with mRNAs (B). Expression profiles of csp genes were also determined by quantitative PCR (C) normalized taking as 100% the level of cspA mRNA at 5 min. Aliquots (1 μg) of total RNA, purified by NucleoSpin RNA II (Macherey-Nagel) from E. coli MRE600 cells growing at 37 °C and harvested at the indicated times, were incubated with DNase I (Sigma) prior to cDNA synthesis by RevertAid First Strand cDNA Synthesis kit (Fermentas). Comparative PCR was performed using the Brilliant SYBR Green QPCR Master Mix (Stratagene) and the following primer pairs: G503 and #70 for cspA; G531 and G532 for cspB; G533 and #221 for cspG ; G534 and #897 for cspI; G506 and G507 for the housekeeping gene dnaG .
A list of oligonucleotides is provided in Table S1.

## Slide 7
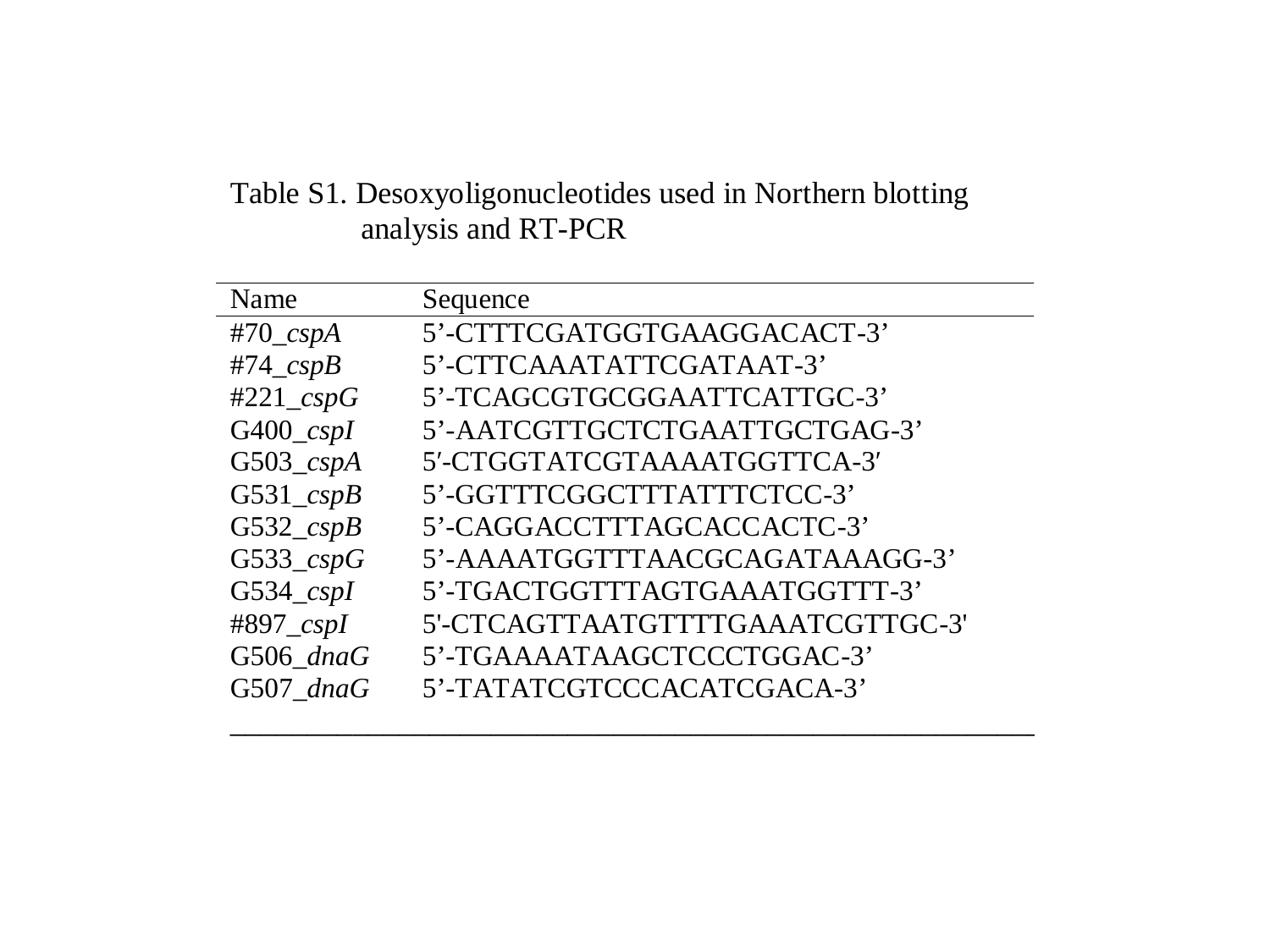

Supplement: Supplementary file 1 [file Presentation1.PPTX]
